# Bilateral inguinal hernia repair by laparoscopic totally extraperitoneal (TEP) vs. laparoscopic transabdominal preperitoneal (TAPP)

**DOI:** 10.1186/s12893-023-02177-2

**Published:** 2023-09-06

**Authors:** Nils Jimmy Hidalgo, Salvador Guillaumes, Irene Bachero, Eugenia Butori, Juan José Espert, César Ginestà, Óscar Vidal, Dulce Momblán

**Affiliations:** 1 Department of Gastrointestinal Surgery, Institute of Digestive and Metabolic Diseases, Hospital Clínic Barcelona, C. de Villarroel, 170, 08036 Barcelona, Spain; 2 Department of General and Digestive Surgery, Institute of Digestive and Metabolic Diseases, Hospital Clínic Barcelona, Barcelona, Spain

**Keywords:** Bilateral inguinal hernia, Laparoscopic, Totally extraperitoneal, Transabdominal preperitoneal

## Abstract

**Background:**

The guidelines recommend laparoscopic repair for bilateral inguinal hernia. However, few studies compare the totally extraperitoneal (TEP) and transabdominal preperitoneal (TAPP) techniques in bilateral inguinal hernias. This study aimed to compare the outcomes of TEP and TAPP in bilateral inguinal hernia.

**Methods:**

We conducted a retrospective cohort study of patients operated on for bilateral inguinal hernia by TEP and TAPP repair from 2016 to 2020. Intraoperative complications, operative time, acute postoperative pain, hospital stay, postoperative complications, chronic inguinal pain, and recurrence were compared.

**Results:**

A total of 155 patients were included in the study. TEP was performed in 71 patients (46%) and TAPP in 84 patients (54%). The mean operative time was longer in the TAPP group than in the TEP group (107 min vs. 82 min, *p* < 0.001). The conversion rate to open surgery was higher in the TEP group than in the TAPP group (8.5% vs. 0%, *p* = 0.008). The mean hospital stay was longer in the TAPP group than in the TEP group (*p* < 0.001). We did not observe significant differences in the proportion of postoperative complications (*p* = 0.672), postoperative pain at 24 h (*p* = 0.851), chronic groin pain (*p* = 0.593), and recurrence (*p* = 0.471). We did not observe an association between the choice of surgical technique (TEP vs. TAPP) with conversion rate, operative time, hospital stay, postoperative complications, chronic inguinal pain, or hernia recurrence when performing a multivariable analysis adjusted for the male sex, age, BMI, ASA, recurrent hernia repair, surgeon, and hernia size > 3cm.

**Conclusions:**

Bilateral inguinal hernia repair by TEP and TAP presented similar outcomes in our study.

## Introduction

Inguinal hernia occurs in 1 to 5% of the general population, they comprise 75% of all abdominal wall hernias [1], and their repair is one of the most performed surgical procedures [2]. Inguinal hernia surgery remains one of the greatest challenges in surgical pathology due to its high frequency and the socioeconomic consequences of even minor complications.

The development of laparoscopic inguinal hernia repair techniques as an alternative to conventional open surgery has improved results such as less postoperative pain, shorter hospital stays, and faster recovery [3, 4]. Some meta-analyses have also shown a lower incidence of chronic pain [5, 6]. One of the reasons for the lower postoperative pain would be the lower rate of complications described in the laparoscopic approach [7]. Randomized trials have found no difference in the recurrence rate between the laparoscopic and open approaches [8, 9].

Studies examining the costs of laparoscopic inguinal hernia compared to a conventional open repair show a higher cost of laparoscopic repair [10, 11]. However, from a socioeconomic perspective and considering the quality-of-life analyses, a laparoscopic procedure is probably the most cost-effective approach for patients in the labor market, especially for bilateral hernias [12].

International guidelines recommend laparoscopic repair for bilateral inguinal hernia [13–16]. The advantage of the laparoscopic approach over the open technique is its ability to address both groins through the same incisions required for unilateral hernia repair. Furthermore, laparoscopy represents a cost-effective procedure compared to the open repair of bilateral inguinal hernia [17].

The most used laparoscopic techniques for inguinal hernia repair are totally extraperitoneal (TEP) and transabdominal preperitoneal (TAPP) [18, 19]. In recent decades, many studies have been performed comparing both laparoscopic approaches with conflicting results. Most previous randomized trials and meta-analyses did not report significant differences in outcomes such as total complications, time back to work, or recurrence rate [20–23]. In contrast, a recent meta-analysis showed less postoperative pain, shorter hospital stays in TEP repair, and shorter duration of surgery in TAPP repair [24]. However, these results were based on studies of unilateral inguinal hernias. Currently, few studies compare the two procedures in bilateral inguinal hernia.

This study aims to evaluate the outcomes of TEP and TAPP in bilateral inguinal hernia repair in our hospital and to compare the intraoperative complications, operative time, acute postoperative pain, hospital stay, postoperative complications, chronic inguinal pain, and hernia recurrence.

## Methods

### Study design

We conducted a retrospective cohort study of patients who underwent bilateral inguinal hernia repair at the Hospital Clinic Barcelona, Spain, a tertiary hospital. Data were obtained by reviewing computerized medical records. The transition from open inguinal hernia repair to laparoscopic repair in our hospital began in 2016.

### Study population

Inclusion criteria: patients who underwent bilateral inguinal hernia repair by laparoscopic approach from January 1, 2016, to December 31, 2020. Exclusion criteria: emergency surgeries, patients without a postoperative follow-up of at least one year, and patients with incomplete data.

### Groups to analyze

The participants in our study were divided into two groups based on the laparoscopic technique used: the TEP group and the TAPP group. Two separate teams performed the procedures. One of the teams, made up of two surgeons, used the TEP technique during the research period, while the other team, made up of three surgeons, used the TAPP approach. At the beginning of our study, the surgeons on both teams had previous experience in laparoscopic surgery but no previous experience in laparoscopic inguinal hernia surgery.

### Totally extraperitoneal technique

An infra-umbilical incision was made up to the opening of the anterior rectus muscle fascia. The anterior rectus muscle was retracted laterally to access the retro muscular space. A balloon trocar was introduced to dissect the retro muscular space until accessing the preperitoneal space, and subsequently, an 11-mm trocar was introduced for a 30-degree laparoscopy camera. An 11-mm trocar and a 5-mm trocar were introduced as working ports in the sub-umbilical midline. Cooper's ligament was exposed, and the peritoneum of the direct or indirect hernia sac was reduced, identifying the spermatic duct or round ligament and gonadal vein, and total exposure of the myopectineal orifice was achieved. A 10 × 15 cm self-adhesive polypropylene mesh was placed. The same procedure was performed for the contralateral hernia.

### Transabdominal preperitoneal technique

Pneumoperitoneum was performed using a Veress needle inserted through a supraumbilical incision. Three trocars were introduced: an 11-mm supraumbilical trocar for the 30-degree laparoscopy camera, an 11-mm trocar on the right flank, and a 5-mm trocar on the left flank as working ports. An incision was made in the peritoneum from the level of the iliac crest to the inguinal ligament. Cooper's ligament was exposed, direct or indirect hernial sac was reduced, identifying the spermatic duct or round ligament and gonadal vein, and total exposure of the myopectineal orifice was achieved. In large direct hernias, seroma prophylaxis was performed with fixation of the redundant transversalis fascia with a 00 barbed suture. A polypropylene mesh of a minimum size of 12 × 15 cm was placed and fixed with tissue adhesive. The closure of the peritoneum was performed with 00 absorbable barbed suture. The same procedure was performed for the contralateral hernia.

### Variables analyzed

We collected patient demographics such as age, gender, and body mass index (BMI). Also, previous comorbidities such as arterial hypertension, heart disease, chronic lung disease, kidney disease, liver disease, diabetes, obesity, smoking history, and lower abdominal surgery were collected. The ASA (American Society of Anaesthesiologists) classification quantified the anesthetic risk.

The hernia characteristics analyzed were the pre-surgical clinical or radiological diagnosis of recurrent hernia. The size of the hernia collected is according to the classification of the European Hernia Society, which classifies hernias into three grades: grade I (< 1.5 cm), grade II (1.5 -3 cm), and grade III (> 3 cm). In our study, we defined hernia size considering the side with the largest hernia according to the finding during surgery.

We also collected the proportion of patients who underwent day surgery, defined as surgery that did not require an overnight hospital stay.

The intraoperative results analyzed were surgical time, intraoperative complications, and conversion to open surgery.

The postoperative results analyzed were complications such as hematoma, urinary retention, urinary infection, seroma, or surgical wound infection. The postoperative complications were classified according to the Clavien-Dindo classification. The length of hospital stay was defined as the time elapsed from admission to discharge. We collected the postoperative pain 24 h after surgery measured from 0–10 cm according to a visual analog scale (VAS). We also collected the readmission to the hospital within 30 days post-surgery; chronic inguinal pain, defined as persistent inguinal pain three months after surgery, measured by a visual analog scale. The hernia recurrence was diagnosed by physical examination or ultrasound in the postoperative follow-up for one year.

### Statistical analysis

Qualitative variables were analyzed using the chi-square test or Fisher's exact test. When analyzing the quantitative variables, we performed a normality test; if the sample was normal, the student's t-test was used, and for non-normal samples, we used the Mann–Whitney U test.

To compare the progression of the surgical times of both groups (TEP and TAPP), we performed a logistic regression analysis of the consecutive cases in the period evaluated.

We performed a multivariable analysis using logistic regression to determine the association of the choice of surgical technique (TEP vs. TAPP) with conversion to open surgery, the presence of any postoperative complication, chronic inguinal pain, and recurrent hernia, adjusted for the male sex, age ≥ 65 years, BMI ≥ 30, ASA III-IV, recurrent hernia repair, surgeon, and grade III hernia size (> 3cm) according to the European Hernia Society classification. For the analysis of quantitative dependent variables such as surgical time or hospital stay, we performed a multiple linear regression analysis.

Statistical analysis was performed using SPSS version 20.0 software (IBM Corp. in Armonk, NY), and we established the statistical significance at *p* < 0.05.

### Ethics

This retrospective database and study were approved by the Ethics Committee of our hospital (HCB/2022/1015).

## Results

### Trends in the use of the laparoscopic approach

During the period studied, 255 patients underwent bilateral inguinal hernia repair. Surgeries were performed by open approach in 95 patients (37.9%) and by laparoscopic approach in 160 patients (62.7%). We observed an increase in the choice of the laparoscopic approach from 22% in 2016 to 94% in 2020 (*p* < 0.001).

**Fig. 1:**
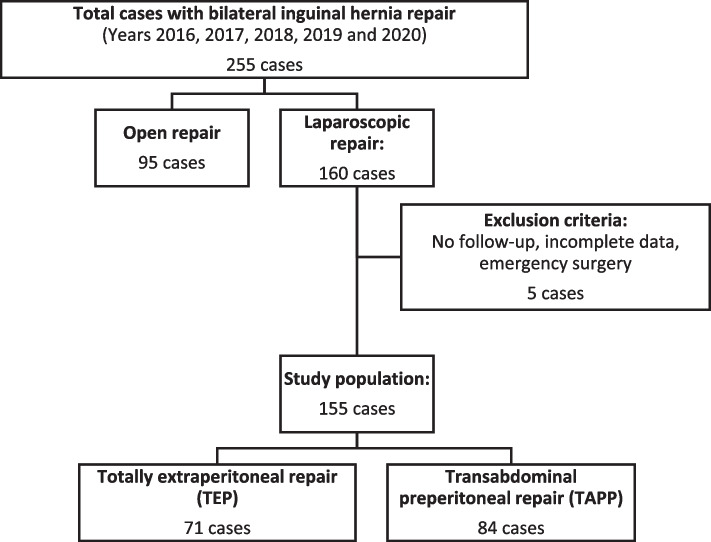
Case selection flow chart

After applying the inclusion and exclusion criteria, 155 patients who underwent laparoscopic bilateral inguinal hernia repair were included. TEP was performed in 71 patients (46%) and TAPP in 84 patients (54%). The case selection flowchart is described in Fig. 1.

### Demographic, comorbidities, and hernia characteristics

When we analyzed our study population divided by the type of laparoscopic approach used (TEP or TAPP), we found no statistically significant differences in age, comorbidities, BMI, or ASA score (Table 1). The male sex ratio was higher in the TEP group than in the TAPP group (98.6% vs. 86.9%, *p* = 0.007). We found no significant differences in the proportion of recurrent hernias or the size of the hernia between both groups. The proportion of ambulatory surgery was higher in the TEP group than in the TAPP group (*p* < 0.001).

**Table 1:** Characteristics of patients with laparoscopic bilateral inguinal hernia repair (2016–2020)

	Total *N* = 155	TEP *N* = 71 (46%)	TAPP *N* = 84 (54%)	*p*-value
Age, Mean ± SD	63.40 ± 12.07	64.54 ± 12.33	62.44 ± 11.82	0.283
Sex, N (%)				
Male	143 (92.3)	70 (98.6)	73 (86.9)	0.007
Female	12 (7.7)	1 (1.4)	11 (13.1)	0.007
Comorbidities, N (%)				
Arterial hypertension	58 (37.4)	27 (38)	31 (36.9)	0.885
Heart disease	15 (9.7)	8 (11.3)	7 (8.3)	0.538
Chronic pulmonary disease	8 (5.2)	5 (7)	3 (3.6)	0.471
Renal disease	8 (8.5)	6 (8.5)	2 (2.4)	0.143
Liver disease	7 (4.5)	3 (4.2)	4 (4.8)	1
Diabetes mellitus	17 (11)	7 (9.9)	10 (11.9)	0.685
Obesity	18 (11.6)	7 (9.9)	11 (13.1)	0.531
Smoking history	80 (51.6)	39 (54.9)	41 (48.8)	0.447
Abdominal lower surgery	33 (21.3)	14 (19.7)	19 (22.6)	0.66
ASA, N (%)				0.347
ASA I	41 (26.5)	18 (25.4)	23 (27.4)	0.775
ASA II	100 (64.5)	44 (62)	56 (66.7)	0.543
ASA III	14 (9)	9 (12.7)	5 (6)	0.146
BMI, Mean ± SD	25.63 ± 3.58	25.19 ± 3.27	25.99 ± 3.8	0.189
Recurrent Repair, N (%)	14 (9)	5 (7)	9 (10.7)	0.427
Hernia size (EHS), n (%)				0.628
Grade I (< 1.5 cm)	15 (9.7)	8 (11.3)	7 (8.3)	
Grade II (1.5–3 cm)	86 (55.5)	39 (54.9)	47 (56)	
Grade III (> 3 cm)	54 (34.8)	24 (33.8)	30 (35.7)	
Outpatient surgery, N (%)	32 (20.6)	29 (40.8)	3 (3.6)	< 0.001

*SD* Standard deviation, *BMI* Body mass index, *ASA* American Society of Anesthesiologists classification, *EHS* European Hernia Society

### Intraoperative and postoperative outcomes

The mean operative time was longer in the TAPP group than in the TEP group (107 min vs. 82 min, *p* < 0.001). The conversion rate to open surgery was higher in the TEP group than in the TAPP group (8.5% vs. 0%, *p* = 0.008). The conversion of the six patients in the TEP group to open surgery was due to technical difficulty in the dissection of the preperitoneal space. Five patients underwent conversion to a Lichtenstein repair, and one patient to a Nyhus repair. Also, one patient converted from TEP to TAPP due to technical difficulty caused by peritoneal rupture. The mean hospital stay was longer in the TAPP group than in the TEP group (*p* < 0.001). We did not observe significant differences in the proportion of specific postoperative complications, complications according to the Clavien-Dindo classification, postoperative pain at 24 h, chronic inguinal pain, or proportion of hernia recurrence (Table 2).

**Table 2:** Outcomes of patients with laparoscopic bilateral inguinal hernia repair (2016–2020)

	Total *N* = 155	TEP *N* = 71 (46%)	TAPP *N* = 84 (54%)	*p*-value
Operative time (min), Mean ± SD	96.11 ± 33.27	82.99 ± 30.84	107.2 ± 31.29	< 0.001
TEP to TAPP conversion, N (%)		1 (1.4)	0 (0)	
Open conversion, N (%)	6 (3.9)	6 (8.5)	0	0.008
Intraoperative complication, N (%)	0	0	0	
Postoperative complications, N (%)				
Hematoma	1 (0.6)	1 (1.4)	0	0.458
Urinary retention or infection	2 (1.3)	1 (1.4)	1 (1.2)	1
Seroma	24 (15.5)	9 (12.7)	15 (17.9)	0.374
Wound infection	1 (0.6)	1 (1.4)	0	0.458
Any complications	27 (17.4)	11 (15.5)	16 (19)	0.672
Clavien Dindo, N (%)				0.143
I	25 (16.1)	10 (14.1)	15 (17.9)	0.525
II	2 (1.3)	1 (1.4)	1 (1.2)	1
Length of stay (days), Mean ± SD	1.05 ± 0.44	0.65 ± 0.61	1.05 ± 0.44	< 0.001
VAS pain 24 h, mean ± SD	2.18 ± 1.6	2.18 ± 0.85	2.17 ± 1.77	0.851
Readmission 30 days, N (%)	0	0	0	
Chronic Inguinal Pain (≥ 3m), N (%)	3 (1.9)	2 (2.8)	1 (1.2)	0.593
Hernia recurrence, N (%)	8 (5.2)	5 (7)	3 (3.6)	0.471

*SD* Standard deviation, *VAS* Visual Analogue Scale

### Evolution of operative time

**Fig. 2:**
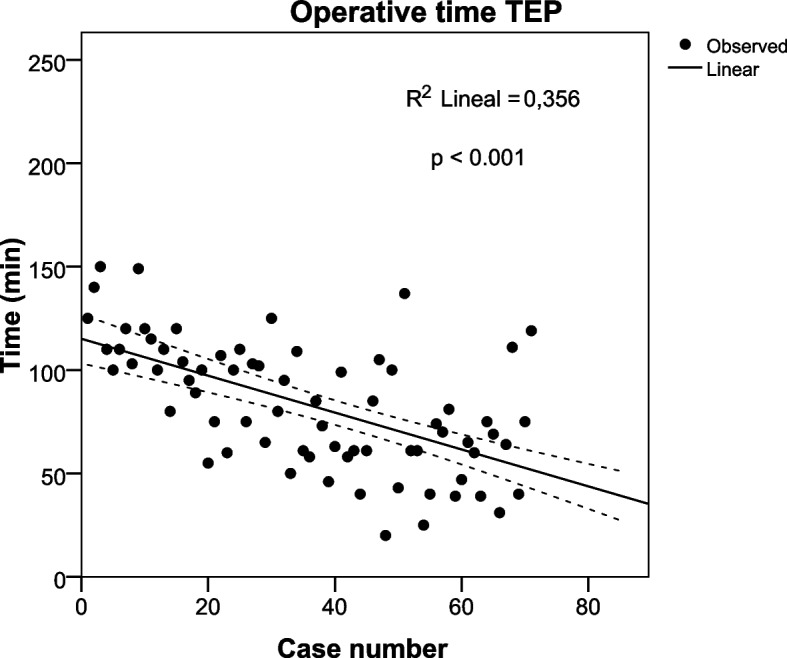
Operative time in consecutive cases of Totally extraperitoneal repair (TEP). Linear regression analysis (R2 = 0.356, *p* < 0.001)

**Fig. 3:**
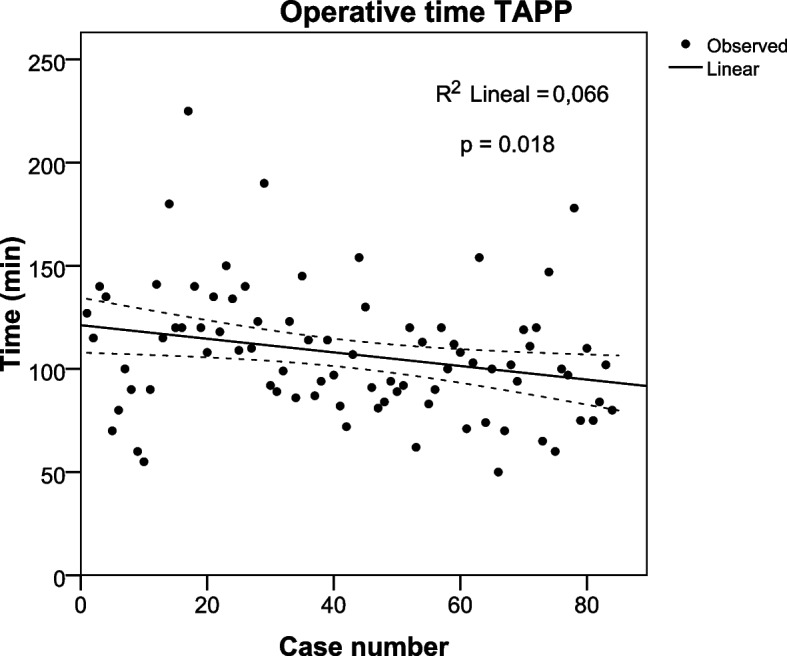
Operative time in consecutive cases of Transabdominal preperitoneal repair (TAPP). Linear regression analysis (R2 = 0.066 *p* = 0.018)

When analyzing the progression of operative time (Figs. 2 and 3) in the study period, through a linear regression analysis, we observed that the decrease in operative time was greater in the TEP group (R2 = 0.356, *p* < 0.001) than in the TAPP group (R2 = 0.066 *p* = 0.018).

### Multivariable analysis of surgical outcomes

When performing a multivariable analysis by logistic regression adjusted for the male sex, age ≥ 65 years, BMI ≥ 30, ASA II-IV, recurrent hernia repair, and hernia size > 3cm, we did not observe an association between the choice of surgical technique (TEP vs. TAPP) with postoperative complications, chronic inguinal pain, or hernia recurrence (Table 3). The differences between both techniques in conversion rate (*p* = 0.999), surgical time (*p* = 0.942), and hospital stay (*p* = 0.381) were not statistically significant.

**Table 3:** Associations between operative method (TEP versus TAPP) and postoperative outcomes of bilateral inguinal hernia repair

TEP versus TAPP	Adjusted Odds^a^	
	OR (95% CI)	*p*-value
Any postoperative complication	0.26 (0.33–2.04)	0.2
Chronic Inguinal Pain	4.42 (0.04–49.93)	0.529
Hernia recurrence	0.89 (0.09–8.3)	0.709

^a^Logistic regression model adjusted for the male sex, age ≥ 65 years, BMI ≥ 30, ASA III-IV, recurrent repair, surgeon, and EHS hernia size III (> 3 cm)

## Discussion

This study did not observe significant differences between TEP and TAPP in conversion rate, operative time, hospital stay, the proportion of postoperative complications, acute postoperative pain, chronic inguinal pain, and hernia recurrence.

The laparoscopic approach in inguinal hernia repair is a valid alternative to traditional open repair [15, 25]. However, despite the recommendations of international guidelines, the utilization rates are variable: 38% in the USA [26], 23% in England [27] and 5.7% in Spain [28]. The use rate of laparoscopy for bilateral inguinal hernia repair in Spain in 2019 was 23% [29].In our study, we observed a significant increase in the use of laparoscopic access for bilateral hernia repair, reaching 94% in 2020.

TEP and TAPP are the two most used laparoscopic procedures for inguinal hernia repair. Most previous studies have not identified advantages between the two laparoscopic techniques [25, 30]. The main difference between TEP and TAPP is the access route to the preperitoneal space. For many groups, TEP is more attractive as it reproduces the access route of the open preperitoneal repair without accessing the abdominal cavity and avoids the risk of intra-abdominal organ injury [3]. In contrast, other groups prefer TAPP as it has an access route more similar to conventional laparoscopy for other pathologies and the advantage of exploring both inguinal regions [31].

Visceral and vascular injuries are the most important intraoperative complications of inguinal hernia repair. It has been described that visceral injuries are more frequent in TAPP than in TEP, reporting an incidence of 0.21% [5, 13]. Vascular lesions, especially inferior epigastric artery lesions, are more common in TEP [32, 33], and 0–3% incidence has been reported [22, 34]. Our study reported no intraoperative complications in bilateral inguinal hernia repairs by TEP and TAPP.

Accidental tears of the peritoneum, bleeding, and adhesions have been reported as the main causes of conversion from laparoscopic repair to open surgery [35, 36]. Previous studies describe a higher incidence of conversion to open surgery in TEP [22, 37]. For anatomical orientation and identification of structures, it is necessary to create an adequate preperitoneal space that allows correct mesh placement and control of complications such as injury to the inferior epigastric artery [37]. The higher conversion rate in TEP could be explained by the greater difficulty in creating and maintaining a wide preperitoneal space, which is worsened by adhesions from previous preperitoneal surgery and tears of the peritoneum [35, 36]. We found six cases of conversion to open surgery in TEP and no conversion in TAPP; however, when performing the multivariable analysis, the technique performed was not associated with the conversion. Conversion to open surgery in the TEP group occurred in cases 1, 10, 15, 25, 31, and 35. These cases were operated on in the first half of the study, possibly related to learning the technique. Previous studies have shown that conversion is greater in the learning phase and that between 30–75 surgeries are required to complete the learning curve [38, 39]. The surgeons who performed TEP and TAPP in our study began and completed their learning curve during the time analyzed; however, this learning was not carried out only in bilateral inguinal hernias. In this same period, the transition to laparoscopic surgery to repair unilateral inguinal hernias also began.

The operative time reported in some studies was longer in TEP [20, 24], while other authors report that the operative time is longer in TAPP [20, 40]. These differences can be explained because the operative time depends on the type of hernia, the patient's condition, and the surgeon's experience [23, 41]. We must remember that these studies were conducted in unilateral hernias; a recent randomized trial in bilateral hernias reported that operative time was longer in TEP [22]. Our study found that the operative time was longer in TAPP; however, these differences were not statistically significant when performing the multivariable analysis. Self-adhering mesh and a balloon dissector to create the preperitoneal space in TEP could decrease operating time. In TAPP, using conventional mesh fixed with glue and subsequent suturing of the bilateral peritoneum increased operating time. In addition, we observed a significant decrease in operative time in both surgical techniques in the study period, which was greater in TEP. At the beginning of the study, the surgical teams had no previous experience in laparoscopic hernia repair; however, the team that performed TEP was made up of two surgeons, and the team that performed TAPP was made up of three surgeons, which could explain the differences.

Differences between TEP and TAPP in common postoperative complications such as hematoma, seroma, wound infection, and urinary retention analyzed in two systematic reviews and meta-analyses were not statistically significant [23, 42]. In a recent meta-analysis, TEP was associated with a lower risk of genital edema, and TAPP repair with a lower risk of seroma formation [43]. A likely explanation could be that TAPP has more surgical space, which facilitates inversion of the transversalis fascia and fixation, associated with a lower incidence of seroma [44]. In our study, the differences in postoperative complications were not significant. When performing multivariable analysis, we observed that the type of technique used was not associated with the presence of complications.

The reported results of the differences in hospital stay between the two techniques are very diverse, probably because it depends on various factors such as age, complication rate, postoperative pain, social factors, educational factors, and trust in the surgeon [45, 46]. A randomized trial found no significant difference in hospital stay between TAPP and TEP in bilateral inguinal hernia repair [22]. In our study, the length of hospital stay was shorter in TEP; however, these differences were not statistically significant when performing the multivariable analysis. The shorter operative time reported in the TEP group could be a favorable factor for the greater use of outpatient surgery in these patients and reduce their hospital stay. The current recommendation is to use outpatient surgery for inguinal hernia repair, regardless of the technique [15]. In recent years, there has been an increase in the percentage of inguinal hernia repairs performed as outpatient surgery [47]. The use of outpatient surgery in inguinal hernia repair is variable in each country, being reported in more than 70% of cases in countries such as Denmark, France, and Sweden [48, 49]. However, it is less than 40% for bilateral inguinal hernias in Spain [29]. The decision of the surgical teams can explain these differences. In our study, the surgeons who performed TAPP used outpatient surgery at the end of the study period. The use of laparoscopy in repairing a bilateral inguinal hernia would increase the use of outpatient surgery by reducing pain and complications compared to open surgery.

Some studies report less early postoperative pain in TEP [21, 24, 50]. However, a recent systematic review found no difference in postoperative pain between TEP and TAPP [51]. Using tacks to fix the mesh and close the peritoneum increases postoperative pain, so glue or self-fixing mesh is recommended [14, 52]. Some authors suggest that postoperative pain is greater in TAPP than in TEP, mainly due to the use of tacks [25, 53]. In our study, self-fixing meshes were used in TEP and glue to fix the mesh in TAPP, and we found no differences in postoperative pain between the two techniques.

Previous studies have found no differences between TEP and TAPP in chronic pain and hernia recurrence [23, 24, 51, 52, 54]. The reported incidence of recurrence of laparoscopic repair is similar to that of open repair [55, 56]. The main causes of recurrence after laparoscopic repair are incomplete dissection, mesh size that is too small, and improper mesh position or migration [57]. Using a mesh of at least 10 × 15 cm, proper surgical technique, and training can significantly reduce the recurrence rate [14, 15]. When we performed a multivariable analysis, we found no association between the type of laparoscopic technique chosen and chronic pain or hernia recurrence.

Previous studies in unilateral and bilateral inguinal hernias have not observed significant differences in the results of TEP and TAPP [22, 23]. However, some studies recommend the TAPP for scrotal hernias and incarcerated hernias [58, 59]. In patients with previous abdominal surgeries, TEP has the advantage of being a procedure completely performed in the preperitoneal space [60]. In our opinion, surgeons can use any of these techniques; however, surgeons from specialized abdominal wall units must know how to perform both techniques.

The limitations of this study are its retrospective design, the small number of cases because we only included bilateral hernias, the performance of laparoscopic techniques (TEP and TAPP) by two different teams of surgeons, the non-assessment of costs and the postoperative follow-up period that was not more than one year. The two groups of surgeons used different synthetic meshes. The choice of mesh depended on the preference of each group of surgeons. Surgeons in the TEP group prioritized reducing surgical time using self-adhesive mesh, while surgeons in the TAPP group used simple polypropylene mesh because of its lower cost. However, its strengths are being one of the few studies that specifically analyzes the results of TEP and TAPP in bilateral inguinal hernia repair, the similarity of the groups analyzed, and the similar experience of surgical teams in inguinal hernia repair by laparoscopy.

## Conclusions

In this study, bilateral inguinal hernia repair by TEP and TAP presented similar conversion rates, operative time, hospital stay, the proportion of postoperative complications, acute postoperative pain, chronic inguinal pain, and hernia recurrence. However, randomized trials are needed to compare the results of both techniques, specifically in bilateral inguinal hernia repair.

## Data Availability

The datasets used and analyzed during the current study are available from the corresponding author on reasonable request.

## Abbreviations

ASA: American Society of Anaesthesiologists
BMI: Body mass index
TEP: Totally extraperitoneal
TAPP: Transabdominal preperitoneal
